# High Familial Risk of Atrial Fibrillation/Atrial Flutter in Multiplex Families: A Nationwide Family Study in Sweden

**DOI:** 10.1161/JAHA.112.003384

**Published:** 2013-02-22

**Authors:** Bengt Zöller, Henrik Ohlsson, Jan Sundquist, Kristina Sundquist

**Affiliations:** 1 Center for Primary Health Care Research, Region Skåne/Lund University, Malmö, Sweden (B., H.O., J.S., K.S.); 2 Stanford Prevention Research Center, Stanford University School of Medicine, Stanford, CA (J.S., K.S.)

**Keywords:** atrial fibrillation, atrial flutter, family history, risk factors, genetics

## Abstract

**Background:**

Although the heritability of atrial fibrillation/atrial flutter (AF/AFl) has been determined, the familial risk in multiplex families is unclear. The main aim of this nationwide study was to determine the familial risk of AF/AFl in multiplex families.

**Methods and Results:**

We examined the familial risk of AF/AFl in the entire Swedish population. We linked Multigeneration Register data on individuals aged 0 to 76 years with Hospital Discharge Register data for 1987–2008 and Outpatient Register data for 2001–2008 to compare AF/AFl risk among relatives of all 300 586 individuals with AF/AFl with that among relatives of unaffected individuals. We used conditional logistic regression to investigate differences in exposure between cases and controls. Parents (odds ratio [OR] 1.95 [95% CI 1.89 to 2.00]) and siblings (OR=3.08 [3.00 to 3.16]) of cases had higher odds of AF/AFl than did parents and siblings of controls. AF/AFl ORs were increased in both sexes. For 2% of cases, both parents had AF/AFl, compared with only 0.7% of controls (OR=3.60 [3.30 to 3.92]). Moreover, 3% of cases had ≥2 siblings with AF/AFl, compared with 1% of controls (OR=5.72 [5.28 to 6.19]). In premature cases (diagnosed at age <50 years), the ORs were 5.04 (4.36 to 5.82) and 8.51 (6.49 to 11.15) for AF/AFl in both parents and AF/AFl in ≥2 siblings, respectively. The overall spouse OR was 1.16 (1.13 to 1.19).

**Conclusions:**

Family history of AF/AFl increases the odds of AF/AFl in first‐degree relatives. High familial risks were observed in multiplex families.

## Introduction

Atrial fibrillation (AF) is a major public health problem because of its increasing prevalence and because it is associated with increased morbidity and mortality.^1^ Familial clustering of AF was first reported in 1943^2^ and has been repeatedly demonstrated since then.^3–11^ The first chromosomal location of an AF susceptibility gene was reported in 1997 based on genetic mapping studies in 3 families.^12^ Several genetic variants have since been linked to the risk of AF.^13–20^

The importance of family history for lone AF has been determined in several studies.^5,7–8,10^ Only 1 nationwide study has been performed—a study of 5269 patients with AF in Iceland (none of whom had atrial flutter [AFl]).^6^ However, 80% of the patients (n=4195) were closely related, being within 4 meioses of another patient. Thus, it is hard to generalize the study's estimated familial risks. Risk of the combined phenotype AF plus AFl has been determined among twins. The hazard ratio (HR) for monozygotic twins compared with dizygotic twins was 2.0.^9^ Two studies from Framingham determined familial risk of AF plus AFl among offspring and siblings.^4,11^ Interestingly, the familial risk was not attenuated by adjustment for risk factors for AF or known AF‐related genetic variants, suggesting that currently known acquired and genetic variants may not fully explain the increased familial risk of AF.^11^ Multiplex families containing ≥2 affected probands are an efficient source of information about high‐risk cases.^21^ In a study from the Framingham Heart Study, AF risk increased as the number of affected first‐degree relatives increased (HR 1.24 [95% CI, 1.05 to 1.46]).^11^ However, the number of multiplex families was limited.^11^ Thus, a firm estimate of familial risk has not been determined for multiplex families with 2 affected parents or ≥2 affected sibling probands in a large‐scale study. Moreover, the risk of AF among spouses, which may reflect the sharing of nongenetic familial factors,^22–23^ has not been previously determined.

It is well established that AF and AFl are clinically related.^24^ Although they may occur in isolation, AF1 often accompanies AF. Electrophysiological studies (see review by Waldo^24^) have advanced understanding of this relationship. AF of varying duration precedes the onset of AFl in most cases. It is therefore not surprising that family history of AF is common in patients with AFl.^7^ Although the familial occurrence of AFl has been reported,^25^ the familial risk of AF1 has not been determined. However, a genome‐wide association study showed that 2 genetic variants on chromosome 4q25 associated with AF (rs2200733 and rs10033464) are also risk factors for AFl.^13^ Thus, it seems justified to study the importance of family history of both AF and AFl, and not just AF.^4,9,11^

In this nationwide study, the odds ratio (OR) for AF/AFl was determined in multiplex families with 2 affected parents or ≥2 affected sibling probands. Moreover, to investigate the contribution of shared environments, spouse risks were assessed.

## Methods

To assess AF/AFl among individuals in Sweden, comprehensive registers and health care data from multiple nationwide sources were linked.^26–29^ This linking was based on unique individual Swedish 10‐digit personal ID numbers assigned at birth or immigration to all Swedish residents for life, information on which is nearly 100% complete. These numbers were replaced with serial numbers to preserve anonymity. Our database contains data from 5 sources:

1. The Swedish Multigeneration Register, which contains information on family relationships (eg, siblings, parent–offspring). The register contains information on index persons registered in Sweden between January 1, 1961, and December 31, 2008, and born between January 1, 1932, and December 31, 2008.
2. The Total Population Register, which contains annual data on education and marital status from 1990 to 2008.
3. The Swedish Hospital Discharge Register, which contains all hospital diagnoses for all people in Sweden from 1987 to 2008. Every record includes the main discharge diagnosis.
4. The Outpatient Care Register, which contains information from all outpatient clinics in Sweden from 2001 to 2008.
5. The Swedish Cause of Death Register, which contains data on date of death from 1961 to 2008. Statistics Sweden and the National Board of Health and Welfare provided the data for the analyses in the present study.

This study was approved by the Ethics Committee of Lund University, Sweden.

### Variable Definition

Cases of AF/AFl in the Swedish Hospital Discharge Register and Outpatient Care Register were identified by *International Classification of Diseases* (ICD) codes 427D (ICD‐9) and I48 (ICD‐10). The same ICD codes were used for AF and AFl until 1997, which prevents separation of these 2 atrial arrhythmias; from 1997 onward, individual subcodes have been used for AF and AF1 (Table 1). The validity of the diagnosis of AF has been evaluated, and diagnoses were found to be correct in 97% of cases in the Hospital Discharge Register.^27,30^ Diagnoses of other cardiovascular disorders such as stroke and myocardial infarction also have ≈95% validity.^27^ Generally, the validity in the Hospital Discharge Register is ≈85% to 95%.^27^

**Table 1.** Descriptive Statistics for AF/AFl in the Swedish Population for Individuals for Whom ICD Subcodes Were Available (1997–2008)

	No., %
AF only (I48.9A, B)	90 187 (90.3)
AFl only (I48.9C, D)	5386 (5.4)
AF and AFl (I48.9E, F)	4273 (4.3)

AF/AFI indicates atrial fibrillation/atrial flutter; ICD, *International Classification of Diseases*.

### Sample

These analyses were based on a database containing information on all 300 586 probands diagnosed with AF/AFl during 1987–2008. The basic characteristics of the inpatients and outpatients are presented in Table 2. Seventy‐five percent of the cases were found in the Hospital Discharge Register. We used a case–cohort approach to investigate our research question. Each case was matched to 5 controls chosen from the total population. This method has been successfully used in previous studies.^28–29^

**Table 2.** Basic Characteristics of Patients With AF/AFl Included in the Hospital Discharge Register (Inpatients) and the Outpatient Register

	AF/AFl Patients in the Hospital Discharge Register*	AF/AFl Patients Not Included in the Hospital Discharge Register (Outpatients)
Sex (females), %	48	39
Age, y (SD)	71.9 (12.9)	70.5 (12.7)
Heart failure, %	28	20
CHD, %	29	24
Level of education, %		
Low <10 y	71	62
Middle 10–12 y	18	24
High >12 y	9	14

AF/AFI indicates atrial fibrillation/atrial flutter; CHD, coronary heart disease.

* Some patients with AF/AFl were included in both the Hospital Discharge Register and the Outpatient Register.

### Statistical Methods

We conducted 5 main analyses. In the first analysis, a parent–offspring analysis (data set I), we analyzed all AF/AFl proband cases whose parents both lived in Sweden sometime between 1987 and 2008. Five controls were randomly chosen from individuals who lived in Sweden at the time of the case's diagnosis of AF/AFl and who were not diagnosed with AF/AFl before the time of the case's AF/AFl diagnosis. This means that a control individual could later be diagnosed with AF/AFl. This approach is conservative: if controls who were later diagnosed with AF/AFl had been excluded from the control group, we might have overestimated the risk of AF/AFl. Furthermore, both parents of the controls also had to have lived in Sweden sometime during 1987–2008. Controls were matched to cases based on year of birth, sex, country of birth, level of education (in the year before the date of diagnosis), and parental year of birth. In 36 783 individuals with AF/AFl, both parents were alive sometime during 1987–2008. There were 4319 cases who could not be matched with 5 controls and were excluded from the analysis. In total, we studied 32 464 cases in the proband–parent analysis.

Our second and third analyses investigated aggregation of AF/AFl among siblings. We began by selecting all AF/AFl proband cases with ≥1 sibling living in Sweden sometime between 1987 and 2008. Five controls were randomly chosen from individuals who lived in Sweden at the time of the case's diagnosis of AF/AFl, and who were not diagnosed with AF/AFl before the time of the case's AF/AFl diagnosis. Controls were matched to cases based on year of birth, sex, country of birth, level of education (in the year before the date of diagnosis), number of siblings who lived in Sweden sometime during 1987–2008, number of male siblings who lived in Sweden sometime during 1987–2008, sibling follow‐up time, and birth order. There were 60 289 individuals with AF/AFl who had ≥1 sibling who was alive during 1987–2008; 4204 cases could not be matched to 5 controls and were excluded from the analysis. In total, we studied 56 085 cases in the first proband–sibling analysis in families with ≥2 siblings (data set II). In the second proband–sibling analysis (data set III), we only studied families with ≥3 siblings (30 691 cases). In a fourth analysis, we merged data sets I and II and only analyzed individuals who were included in both data sets (17 737 cases) (data set IV).

Our fifth analysis investigated aggregation of AF/AFl among spouses (data set V). We began by selecting all AF/AFl proband cases during 1991–2008 who were registered as married in the year before their AF/AFl diagnosis. Five controls were randomly chosen from individuals who lived in Sweden at the time of the case's diagnosis of AF/AFl and who were not diagnosed with AF/AFl before the time of the case's AF/AFl diagnosis. Controls were matched based on year of birth, sex, country of birth, level of education (the year before the date of diagnosis), spouse follow‐up time, and spouse year of birth. Furthermore, the controls had to be registered as married in the year before the case's diagnosis of AF/AFl. In total, 126 302 individuals with AF/AFl were registered as married the year before their diagnosis of AF/AFl. There were 15 700 cases who could not be matched to 5 controls and were excluded from the analysis. We thus studied a total of 110 602 cases in the spouse analysis.

We used conditional logistic regression to investigate the difference in exposure between cases and controls. For the proband–parent analysis, we defined the exposure variable as a categorical variable: 0/1/2 parents affected by AF/AFl. For the first proband–sibling analysis, the exposure variable was first defined as a categorical variable: 0/1+ siblings affected by AF/AFl. The exposure variable for the second proband–sibling analysis was also a categorical variable: 0/1/2+ siblings affected by AF/AFl. We used 0 as the reference category in all analyses.

To take into account the nonindependence of observations from the same family, we used a robust sandwich estimator.^28–29^ For all data sets, we performed 5 analyses (1 to 5): (1) all cases; (2) male cases; (3) female cases; (4) cases who were younger than 50 at diagnosis; and (5) cases who were 50 years or older at diagnosis.

We present ORs and corresponding 95% CIs.^31^ ORs are to be interpreted as follows: an OR of 1.5 implies that the odds are 50% higher than in the corresponding control group. All calculations were performed using SAS version 9.2 (SAS Institute).

## Results

In the entire study population, there were 3 204 349 families with 2 siblings (Table 3). In 23 972 of the 2‐sibling families, 1 sibling had AF/AFl; in 1001 of the 2‐sibling families, both siblings had AF/AFl. Thus, 92% of the sibling cases in 2‐sibling families were from families with only 1 affected sibling and 8% from families with 2 affected siblings. Table 3 also shows that the proportion of cases from families with higher numbers of affected siblings increased with number of siblings in the family.

**Table 3.** Description of the Swedish Population During the Study Period, Showing Numbers of Families With 2, 3, and 4+ Siblings and Numbers of Siblings With AF/AFl

No. of Siblings in Family	No. of Families				Percentage of of Cases from Families*		
	0 Siblings With AF/AFl	1 Sibling With AF/AFl	2 Siblings With AF/AFl	3+ Siblings With AF/AFl	Cases from Families With 1 Affected Sibling	Cases from amilies With 2 Affected Siblings	Cases from Families With 3+ Affected Siblings
2	3 179 376	23 972	1001	NA	92	8	NA
3	621 490	14 861	1036	52	87	12	1
4+	256 964	12 077	1635	268	76	19	5

AF/AFI indicates atrial fibrillation/atrial flutter; NA, not applicable.

* Percentage of cases from families with 2, 3, and 4 or more siblings with 1, 2, or 3 or more affected siblings, respectively.

### Familial Risk of AF/AF1 in Parents of Affected Offspring (Data Set I)

There were 32 464 individuals with AF/AFl, all of whose parents were alive sometime during the study period, who could be matched to 5 controls (Table 4). The mean age of the cases was 52 years. Twenty‐three percent of cases had 1 parent with AF/AFl, compared with 13.6% of controls. Regression analysis gave an OR of 1.95 (95% CI 1.89 to 2.00), which can be interpreted as an almost 2‐fold increase in the odds of AF/AFl in parents of individuals diagnosed with AF/AFl compared with the parents of controls. For 2% of cases, both parents had AF/AFl, compared with only 0.7% of controls (OR 3.60 [95% CI 3.30 to 3.92]). The OR for AF/AFl in both parents was significantly higher than the OR for AF/AFl in 1 parent, indicating that the risk of AF/AFl was higher in individuals with both parents diagnosed with AF/AFl compared with those with only 1 parent with AF/AFl. The ORs were higher in male subjects compared with female subjects, but the CIs in the analyses of AF/AFl in 1 parent overlapped. Analysis of cases with premature AF/AF1 (diagnosis before 50 years of age) gave ORs of 2.33 and 5.04 for AF/AFl in 1 and 2 parents, respectively. Compared with cases with later onset (diagnosis after 49 years of age), the ORs for AF/AFl in parents were higher for cases with premature AF/AFl. Further analysis showed a 2‐fold increase in the odds of AF/AFl in the mothers of cases compared with the mothers of controls (Table 5). The corresponding OR for fathers was 1.85 (95% CI 1.79 to 1.91). Although the ORs for AF/AFl were similar for the mothers of female and male cases (OR 2.04 [95% CI 1.91 to 2.17] and 2.01 [95% CI 1.94 to 2.09], respectively), the OR for AF/AFl in fathers of female cases was slightly lower than the OR for fathers of male cases (OR 1.67 [95% CI 1.56 to 1.79] versus 1.91 [95% CI 1.84 to 1.99]).

**Table 4.** Descriptive Statistics/Results From Conditional Logistic Regression Analysis of Familial Risk of AF/AFl in the Swedish Population (1987–2008): Parents of Probands (Data Set I)

	Cases				Controls			OR (95% CI) for AF/AFl in 1 Parent	OR (95% CI) for AF/AFl in Both Parents
	No.	Mean Age at Diagnosis, y (SD)*	AF/AFl in 1 Parent, %*	AF/AFl in Both Parents, %*	No.	AF/AFl in 1 Parent, %*	AF/AFl in Both Parents, %*		
All	32 464*	52 (13)	7338 (22.6)	640 (2.0)	162 320	22 013 (13.6)	1065 (0.7)	1.95 (1.89–2.00)	3.60 (3.30–3.92)*
Males	23 875	51 (13)	5425 (22.7)	496 (2.1)	119 375	16 207 (13.6)	768 (0.6)	1.96 (1.90–2.02)	3.87 (3.51–4.27)
Females	8589	54 (13)	1913 (22.3)	145 (1.7)	42 945	5806 (13.5)	297 (0.7)	1.91 (1.81–2.01)	2.89 (2.43–3.44)
Age ≤49 y	11 764	38 (9)	2678 (22.8)	248 (2.1)	58 820	6983 (11.9)	311 (0.5)	2.33 (2.23–2.44)	5.04 (4.36–5.82)
Age 50+ y	20 700	60 (6)	4660 (22.5)	392 (1.9)	103 500	15 030 (14.5)	754 (0.7)	1.77 (1.71–1.83)	3.03 (2.72–3.37)

AF/AFI indicates atrial fibrillation/atrial flutter.

* The age is the same for cases and controls.

* Number and percentage of cases and controls who have a parental history (1 or both) of AF/AFl, respectively.

* 61.8% of cases had >1 AF/AFl diagnosis.

* OR for both parents with AF/AFl vs 1 parent with AF/AFl=1.85 (95% CI 1.69–2.02).

**Table 5.** Descriptive Statistics/Results From Conditional Logistic Regression Analysis of Familial Risk of AF/AFl in the Swedish Population (1987–2008): Parents of Probands (Dataset I)

	Number	Odds Ratio (95% CI) for AF/AFl in 1 Parent	Odds Ratio (95% CI) for AF/AFl in Both Parents
Excluding CHD	25 592	2.01 (1.95 to 2.07)	3.70 (3.37 to 4.07)
Excluding HF	29 035	1.97 (1.92 to 2.03)	3.61 (3.30 to 3.95)
Born in Sweden	32 117	1.94 (1.89 to 1.99)	3.61 (3.31 to 3.93)
Born outside Sweden	347	2.94 (2.25 to 3.84)	2.57 (0.84 to 7.86)
Low level of education	8874	1.73 (1.64 to 1.83)	3.51 (2.94 to 4.20)
Middle level of education	14 526	2.00 (1.92 to 2.08)	3.34 (2.93 to 3.81)
High level of education	9064	2.06 (1.96 to 2.17)	4.03 (3.49 to 4.65)
		Odds Ratio (95% CI) for AF/AFl in Mothers	Odds Ratio (95% CI) for AF/AFl in Fathers
All cases	32 464	2.02 (1.95 to 2.09)	1.85 (1.79 to 1.91)
Males	23 875	2.01 (1.94 to 2.09)	1.91 (1.84 to 1.99)
Females	8589	2.04 (1.91 to 2.17)	1.67 (1.56 to 1.79)

AF/AFl indicates atrial fibrillation/atrial flutter; CHD, coronary heart disease; HF, heart failure.

### Familial Risk of AF/AF1 in Siblings of Affected Probands (Data Sets II and III)

There were 56 085 individuals with AF/AFl, with ≥1 sibling who was alive during the study period, who could be matched to 5 controls (Table 6). The mean age of the cases was 57 years. Fifteen percent of cases had ≥1 sibling with AF/AFl, compared with 6% of controls. The OR from the conditional logistic regression analysis was 3.08 (95% CI 3.00 to 3.16), which can be interpreted as an almost 3‐fold increase in the odds of AF/AFl in individuals with ≥1 sibling diagnosed with AF/AFl compared with those with no siblings diagnosed with AF/AFl. The OR was higher in female cases compared with male cases, but the 95% CIs overlapped. The OR for the siblings of individuals with premature AF/AF1 (diagnosis before 50 years of age) was 4.08 (95% CI 3.79 to 4.41), which was higher than for siblings of cases with later onset of AF/AFl (diagnosis after 49 years of age).

**Table 6.** Descriptive Statistics and Results From Conditional Logistic Regression Analysis of AF/AFl in the Swedish Population (1987–2008): Siblings in Families With ≥2 Siblings (Data Set II)

	Cases			Controls	AF/AFl in ≥1 Sibling, %*	OR (95% CI) for AF/AFl in ≥1 Sibling
	No.	Mean Age at Diagnosis, y (SD)*	AF/AFl in ≥1 Sibling, %*	No.		
All	56 085*	57 (11)	8247 (14.7)	280 425	15 763 (5.6)	3.08 (3.00–3.16)
Males	39 648	56 (11)	5555 (14.0)	198 240	10 794 (5.4)	3.01 (2.92–3.11)
Females	16 437	59 (11)	2692 (16.4)	82 185	4969 (6.1)	3.23 (3.09–3.38)
Age ≤49 y	12 125	39 (9)	986 (8.1)	60 625	1412 (2.3)	4.08 (3.79–4.41)
Age 50+ y	43 960	61 (6)	7261 (16.5)	219 800	14 351 (6.5)	2.98 (2.90–3.06)

AF/AFI indicates atrial fibrillation/atrial flutter.

* The age is the same for cases and controls.

* Number and percentage of cases and controls who have a sibling history (≥1 affected sibling) of AF/AFl, respectively.

* 63.7% of cases had >1 AF/AFl diagnosis.

There were 30 691 individuals with AF/AFl, with ≥2 siblings who were alive during the study period, who could be matched to 5 controls (Table 7). The mean age of the cases was 57 years. Sixteen percent of cases had 1 sibling with AF/AFl, compared with 7% of controls; 3% of cases had ≥2 siblings with AF/AFl, compared with 1% of controls. The OR for AF/AFl in ≥2 siblings was 5.72, indicating an almost 6‐fold increase in the odds of AF/AFl in individuals with ≥2 siblings diagnosed with AF/AFl compared with those with no siblings diagnosed with AF/AFl.

**Table 7.** Descriptive Statistics/Results From Conditional Logistic Regression Analysis of AF/AFl in the Swedish Population (1987–2008): Siblings in Families With ≥3 Siblings (Data Set III)

	Cases				Controls			OR (95% CI) for AF/AFl in 1 Sibling	OR (95% CI) for AF/AFl in ≥2 Siblings
	No.	Mean Age at Diagnosis, y (SD)*	AF/AFl in 1 Sibling, %*	AF/AFl in ≥2 Siblings, %*	No.	AF/AFl in 1 Sibling, %*	AF/AFl in ≥2 Siblings, %*		
All	30 691	57 (11)	4970 (16.2)	897 (2.9)	153 455	10 753 (7.0)	986 (0.6)	2.78 (2.69–2.87)	5.72 (5.28–6.19)*
Males	21 584	56 (11)	3377 (15.7)	546 (2.5)	107 920	7374 (6.8)	656 (0.6)	2.73 (2.62–2.84)	5.17 (4.69–5.71)
Females	9107	59 (11)	1593 (17.5)	351 (3.9)	45 535	3379 (7.4)	330 (0.7)	2.89 (2.73–3.06)	6.88 (6.02–7.85)
Age ≤49 y	6360	40 (10)	661 (10.4)	91 (1.4)	31 800	1027 (3.2)	72 (0.2)	3.85 (3.51–4.21)	8.51 (6.49–11.15)
Age 50+ y	24 331	61 (6)	4309 (17.7)	806 (3.3)	121 655	9726 (8.0)	914 (0.8)	2.66 (2.57–2.75)	5.49 (5.06–5.97)

AF/AFI indicates atrial fibrillation/atrial flutter.

* The age is the same for cases and controls.

* Number and percentage of cases and controls who have a sibling (1 or ≥2 affected siblings) history of AF/AFl, respectively.

* OR for 2 siblings with AF/AFl vs 1 sibling with AF/AFl=2.06 (95% CI 1.89–2.24).

The OR for the siblings of ≥2 individuals with premature AF/AF1 (diagnosis before 50 years of age) was 8.51 (95% CI 6.49 to 11.15), which was higher than in siblings of 2 cases with later onset of AF/AFl (diagnosis after 49 years of age).

### Familial Risk of AF/AFl in Individuals With Both Siblings and Parents With AF/AFl (Data Set IV)

The OR for AF/AFl in individuals with ≥1 affected parent and ≥1 affected sibling was 5.56 (95% CI 4.99 to 6.20). This indicates that there was a large increase in the odds of AF/AFl in individuals with ≥1 parent and ≥1 sibling with AF/AFl, compared with those with no siblings or parents diagnosed with AF/AFl (Table 8).

**Table 8.** Results From Conditional Logistic Regression Analysis of Familial Risks of AF/AFl Among Offspring in the Swedish Population (1987–2008): Proband‐Parent‐Sibling (Data Set IV)

	OR (95% CI) for AF/AFl in Siblings Only	OR (95% CI) for AF/AFl in Parents Only	OR (95% CI) for AF/AFl in Both Sibling and Parents
All individuals	3.07 (2.83–3.33)	2.03 (1.96–2.11)	5.56 (4.99–6.20)

AF/AFI indicates atrial fibrillation/atrial flutter.

### Familial Risk of AF/AF1 in Spouses (Data Set V)

The mean age of the spouses at diagnosis of AF/AFl in the cases was 67.4 years, very similar to the mean age of the cases themselves at diagnosis (69 years). The prevalence of AF/AFl was 8.6% among the spouses of cases and 7.5% among the spouses of controls (Table 9). The OR for AF/AFl among the spouses of cases was 1.16, indicating a small increase in the odds of AF/AFl in the spouses of individuals diagnosed with AF/AFl compared with the spouses of individuals not diagnosed with AF/AFl. While the ORs for male and female spouses were similar, the OR for spouses of cases with premature AF/AFl (diagnosis before 50 years of age) was higher than that for spouses of cases with later onset.

**Table 9.** Descriptive Statistics/Results From Conditional Logistic Regression Analysis of AF/AFl in the Swedish Population (1987–2008): Spouses (Data Set V)

	Cases			Controls		OR (95% CI) for AF/AFl in Spouse
	No.	Mean Age at Diagnosis, y (SD)*	AF/AFl in Spouse, %*	No.	AF/AFl in Spouse, %*	
All	110 602*	69 (11)	9458 (8.6)	511 468	41 542 (7.5)	1.16 (1.13–1.19)
Males	66 417	68 (12)	4771 (6.7)	334 933	20 947 (5.9)	1.15 (1.12–1.19)
Females	34 727	70 (10)	4687 (11.9)	176 475	20 595 (10.5)	1.16 (1.12–1.20)
Age ≤49 y	6247	43 (6)	80 (1.3)	30 954	281 (0.9)	1.43 (1.12–1.84)
Age 50+ y	104 355	70 (9)	9378 (9.0)	521 775	41 261 (7.9)	1.16 (1.12–1.18)

AF/AFI indicates atrial fibrillation/atrial flutter.

* The age is the same for cases and controls.

* Number and percentage of cases and controls who have a spouse history of AF/AFl.

* 58.1% of cases had >1 AF/AFl diagnosis.

### Stratified Analysis of Familial Risk

Tables 5 and 10 show the results of the stratified analysis of risk of AF/AFl in the parents and siblings of cases, respectively. Excluding cases with coronary heart disease or heart failure did not significantly affect the odds of AF/AFl. The OR for AF/AFl in ≥1 sibling was higher for cases who were born abroad than for cases who were born in Sweden. The ORs were also higher for the siblings and parents of cases with high levels of education than for the siblings and parents of cases with low levels of education. Among spouses, the ORs were similar for all strata, except for the spouses of cases with high levels of education, who had a higher OR for AF/AFl than the spouses of cases with low levels of education (Table 11).

**Table 10.** Descriptive Statistics/Results From Conditional Logistic Regression Analysis of AF/AFl in the Swedish Population (1987–2008): Siblings in Families With at Least 2 Siblings

	Number of Cases	Odds Ratio (95% CI) for AF/AFl in at Least 1 Sibling
Excluding CHD	37 623	3.27 (3.17 to 3.38)
Excluding HF	47 725	3.14 (3.05 to 3.23)
Born in Sweden	55 774	3.08 (3.00 to 3.16)
Born outside Sweden	311	4.92 (2.87 to 8.44)
Low level of education	19 075	3.01 (2.89 to 3.13)
Middle level of education	23 131	3.12 (3.00 to 3.25)
High level of education	13 879	3.15 (2.98 to 3.33)

AF/AFl indicates atrial fibrillation/atrial flutter; CHD, coronary heart disease; HF, heart failure.

**Table 11.** Results From Conditional Logistic Regression Analysis of AF/AFl in the Swedish Population (1987–2008): Spouses

	Odds Ratio (95% CI) for AF/AFl in Spouses
Excluding CHD	1.16 (1.12 to 1.20)
Excluding HF	1.19 (1.16 to 1.22)
Born in Sweden	1.16 (1.13 to 1.19)
Born outside Sweden	1.12 (1.04 to 1.22)
Low level of education	1.14 (1.12 to 1.17)
Middle level of education	1.21 (1.13 to 1.30)
High level of education	1.33 (1.22 to 1.46)

AF/AFl indicates atrial fibrillation/atrial flutter; CHD, coronary heart disease; HF, heart failure.

## Discussion

The present study is, to our knowledge, the largest nationwide study to estimate the familial risk of AF/AFl. A previous nationwide study from Iceland was smaller and largely focused on closely related patients.^6^ Our study of the Swedish population shows that familial factors are important in the risk of AF/AFl. Our familial risk estimates are similar to those reported from 3 previous studies that estimated familial risks among patients with AF.^4,6,11^ Using a combined AF/AF1 phenotype, we obtained approximately the same risk estimate for first‐degree relatives as that reported in the Icelandic study, which assessed only AF,^6^ and that for the combination of AF and AF1 in the Framingham Heart Study.^4,11^ The results of the present study add to previous AF/AF1 studies by providing firm estimates for multiplex families. Relatives of multiplex probands (≥2 affected probands) are at a high risk of AF/AFl, especially relatives of individuals with onset before the age of 50.

The present study estimated, for the first time, risk of AF/AFl among spouses of individuals with AF/AFl and showed that the nongenetic familial contribution to the observed familial risks was relatively small (Table 9). Spouses may share lifestyle factors such as smoking, alcohol consumption, exercise, and diet to a greater degree than siblings and parent–offspring pairs.^22–23^ Alcohol and smoking have both been suggested to be risk factors for AF.^32–33^ Spouses may also share anthropometric characteristics (eg, body mass index) that may also contribute to increased AF risk.^34^ Interestingly, the risk of AF/AFl was slightly higher among spouses of cases diagnosed with AF/AFl when aged <50 years than the spouse of cases aged 50 or older at diagnosis. The cause of this difference is unclear.

The high risk of AF/AFl in multiplex families may have a genetic basis and suggests the segregation of strong genetic risk factors in multiplex families. The relatively low risk of AF/AFl among the spouses of affected individuals and the higher risk of AF/AFl in the first‐degree relatives of individuals diagnosed with AF/AFl before the age of 50 suggest that the observed familial risks have a stronger genetic than a nongenetic basis. Moreover, even in the face of a complete correlation in exposure among first‐degree relatives, environmental risk factors with relative risks of <10 yield modest familial relative risks (1 to 2) and low recurrence risks.^35^ Similar findings are obtained when familial aggregation of 2 additive environmental factors is considered.^35^ No strong acquired risk factors have yet been described for AF/AFl,^36–37^ suggesting that the high risk of AF/AFl in multiplex families has a considerable genetic basis. Genetic studies could concentrate on multiplex families with prematurely affected individuals, which will increase the probability of finding new genetic variants associated with AF/AF1. Genome scanning of multiplex sibling families may be an important option for identifying genetic risk factors.

The present study has a number of strengths. These include complete nationwide coverage in a country with high medical standards and medical diagnosis of patients by specialists during examinations in hospitals. In addition, the results were not affected by recall bias because they were based on medical diagnoses. Importantly, the Multigeneration Register is a validated source that has been proved to be reliable in the study of many familial diseases.^26–29^

The present study has a number of limitations. The Swedish Hospital Discharge Register contains complete data only since 1987 and the Outpatient Care Register, since 2001. Because of this, we chose to study the 22‐year period between 1987 and 2008. Events that occurred before 1987 are unknown, which most likely creates nondifferential bias regarding familial risk estimates. Moreover, the lack of outpatient data before 2001 is also most likely a source of nondifferential bias in the estimation of familial risks.

Another potential limitation is that we do not have access to the methods used for objective diagnosis. However, the Swedish Hospital Discharge Register has high validity, especially for cardiovascular disorders such as AF, stroke, and myocardial infarction (≈95%).^26–27,30^ We had no data on risk factors for AF/AF1, which are potential confounders. To address this, cases and controls were matched according to educational level, which is related to cardiovascular disease risk factors such as smoking and alcohol intake. Inclusion of both AF and AFl may constitute a limitation. However, AFl is a much less prevalent diagnosis than AF, resulting in a negligible bias in the present study, which included very large numbers of cases and controls (Tables 11 to 3)

The large number of comparisons is another limitation and is a technical point worthy of consideration. Some associations may conceivably have been due to chance, and consistency between the results of this study and other studies, as well as biological plausibility, should be considered when assessing causality.

While not all patients may seek help for AF, affordability of health care is probably not a selective factor in Sweden because of equal access to primary and hospital care. Nor is the likelihood of seeking medical advice likely to be important. The lower spouse correlation and results of stratified analyses do not suggest strong selection bias for hospitalization of certain families.

The incidence of AF/AFl increases with age. Individuals were matched according to age, which was therefore not a source of bias in the present study. We cannot, however, estimate familial risk in individuals older than 76 as the Multigeneration Register contains data only from 1932 onward. However, the results of age‐stratified analyses show that the importance of familial factors decreases with increasing age.

While the present study was limited to Sweden, stratified analysis of familial risks in parents, siblings, and spouses of individuals born outside Sweden gave similar estimates (Tables 5, 10, and 11 which indicates that similar findings might be expected in other populations. However, generalizability to other countries is uncertain. So far, heritability of AF has largely been studied in individuals of European ancestry.^3–11^

## Conclusions

The present study demonstrates that family history of AF/AF1 is an important risk factor for AF/AF1 in the Swedish population. Risk of AF/AFl was especially high in multiplex families and in relatives of individuals diagnosed with AF/AFl before the age of 50. We also report evidence that shared non‐genetic familial factors among spouses have a modest influence on AF/AF1 risk.
